# Publisher Correction: Genome-wide analysis of Corsican population reveals a close affinity with Northern and Central Italy

**DOI:** 10.1038/s41598-019-55185-9

**Published:** 2019-12-06

**Authors:** Erika Tamm, Julie Di Cristofaro, Stéphane Mazières, Erwan Pennarun, Alena Kushniarevich, Alessandro Raveane, Ornella Semino, Jacques Chiaroni, Luisa Pereira, Mait Metspalu, Francesco Montinaro

**Affiliations:** 1 Institute of Genomics, University of Tartu, Tartu, Estonia; 2 Aix Marseille Univ, CNRS, EFS, ADES, Marseille, France; 3 Etablissement Français du Sang PACA Corse, Biologie des Groupes Sanguins, Marseille, France; 4 Institute of Genetics and Cytology, National Academy of Sciences of Belarus, Minsk, 220072 Belarus; 5 Dipartimento di Biologia e Biotecnologie “L. Spallanzani” Università di Pavia, Via Ferrata 9, 27100 Pavia, Italy; 6 i3S - Instituto de Investigação e Inovação em Saúde, Universidade do Porto, 4200-135 Porto, Portugal; 7 Instituto de Patologia e Imunologia Molecular da Universidade do Porto (IPATIMUP), 4200-135 Porto, Portugal; 8 Department of Zoology, University of Oxford, Oxford, UK

Correction to: *Scientific Reports* 10.1038/s41598-019-49901-8, published online 19 September 2019

In the original version of this Article, the author Julie Di Cristofaro was incorrectly indexed.

Additionally, a Supplementary File containing Table S5 was omitted from the original version of this Article.

These errors have been corrected in the HTML version of the Article; the PDF version was correct at time of publication.

