# Supplementary material for: Genome-wide analysis of Corsican population reveals a close affinity with Northern and Central Italy
Source: Sci Rep. 2019 Sep 19;9:13581. doi: 10.1038/s41598-019-49901-8 (PMC6753063; doi:10.1038/s41598-019-49901-8)
Supplement: Supplementary file 1 — Supplementary Figures and Tables [file 41598_2019_49901_MOESM1_ESM.pdf]

# Genome-wide analysis of Corsican population reveals a close affinity with Northern and Central Italy

**Erika Tamm[1], Julie Di Cristofaro[2,3], Stéphane Mazières[2], Erwan Pennarun[1], Alena Kushniarevich[1,4], Alessandro Raveane[5], Ornella Semino[5], Jacques Chiaroni[2,3], Luisa Pereira[6,7], Mait Metspalu[1], Francesco Montinaro[1,8]**

[1] Institute of Genomics, University of Tartu, Tartu, Estonia.

[2] Aix Marseille Univ, CNRS, EFS, ADES, Marseille, France.

[3] Etablissement Français du Sang PACA Corse, Biologie des Groupes Sanguins, Marseille, France.

[4] Institute of Genetics and Cytology, National Academy of Sciences of Belarus, Minsk 220072, Belarus.

[5] Dipartimento di Biologia e Biotecnologie "L. Spallanzani" Università di Pavia, Via Ferrata 9, 27100 Pavia, Italy.

[6] i3S - Instituto de Investigação e Inovação em Saúde, Universidade do Porto, 4200-135 Porto, Portugal.

[7] Instituto de Patologia e Imunologia Molecular da Universidade do Porto (IPATIMUP), 4200-135 Porto, Portugal.

[8] Department of Zoology, University of Oxford, Oxford, UK.

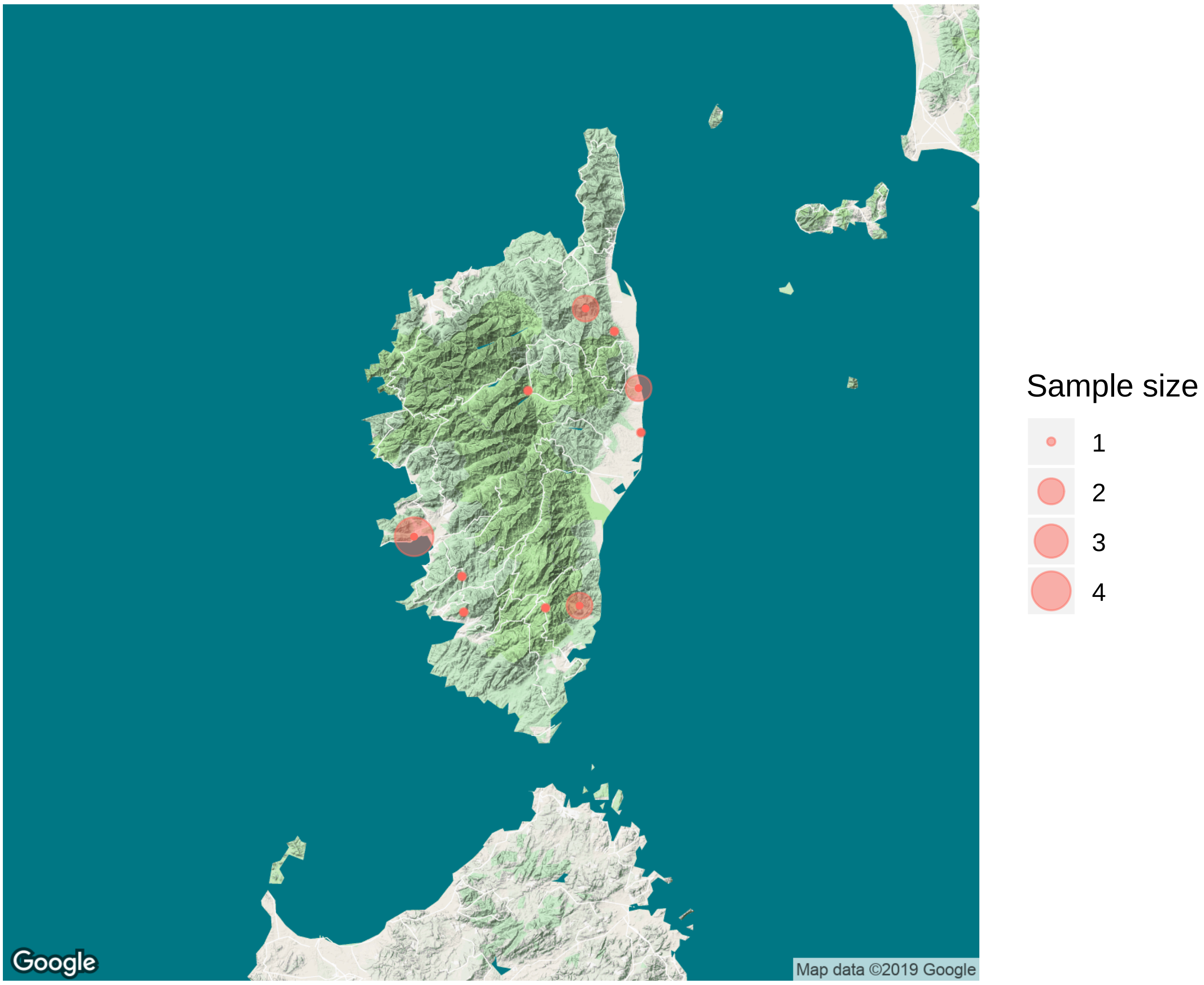


**Supplementary Figure S1.** Map of Corsica Island showing sampling locations and sizes for newly reported Corsican samples. Map data: Google.

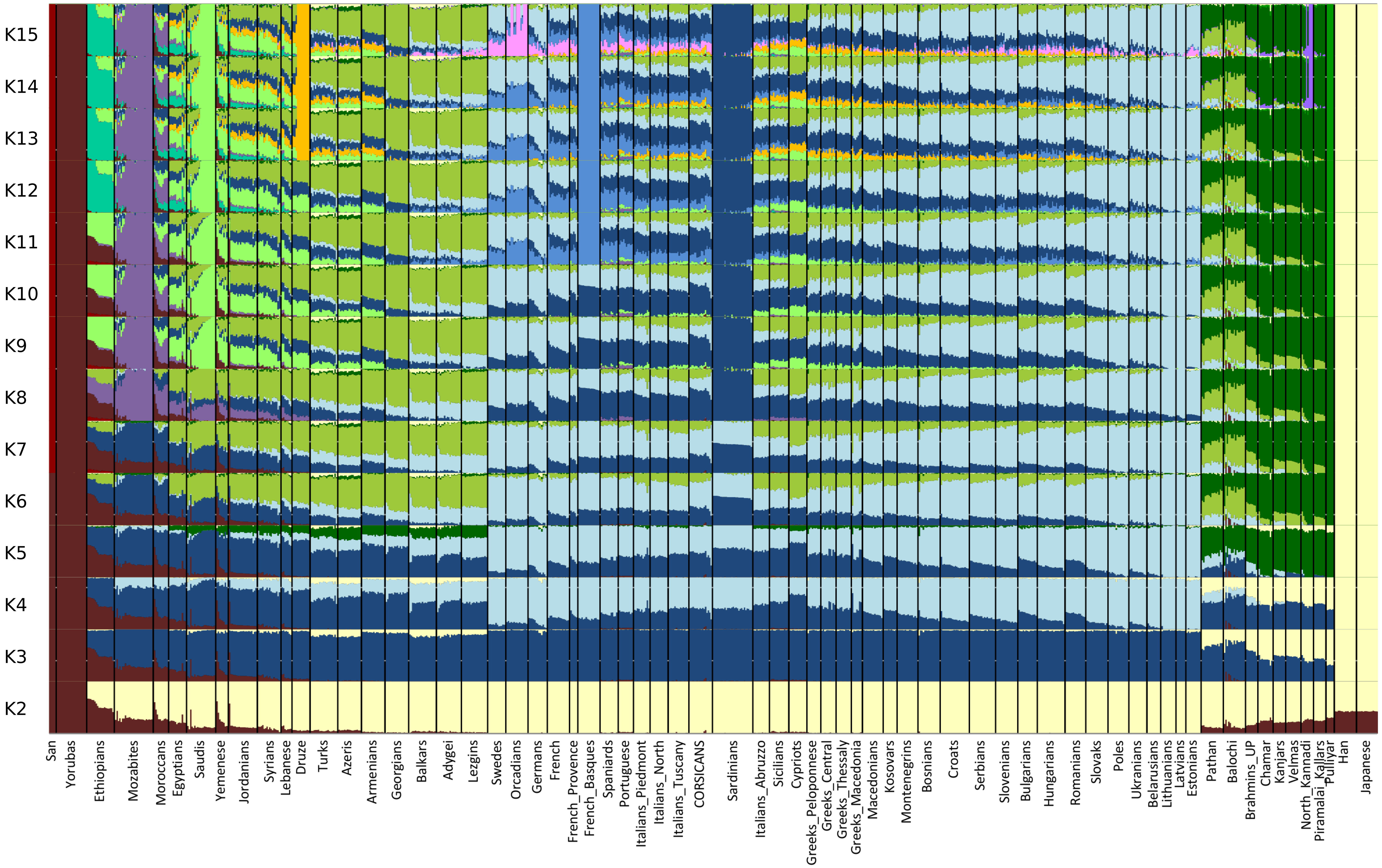


**Supplementary Figure S2.** ADMIXTURE plot of Corsican population in a worldwide context at K =2 to K = 15.

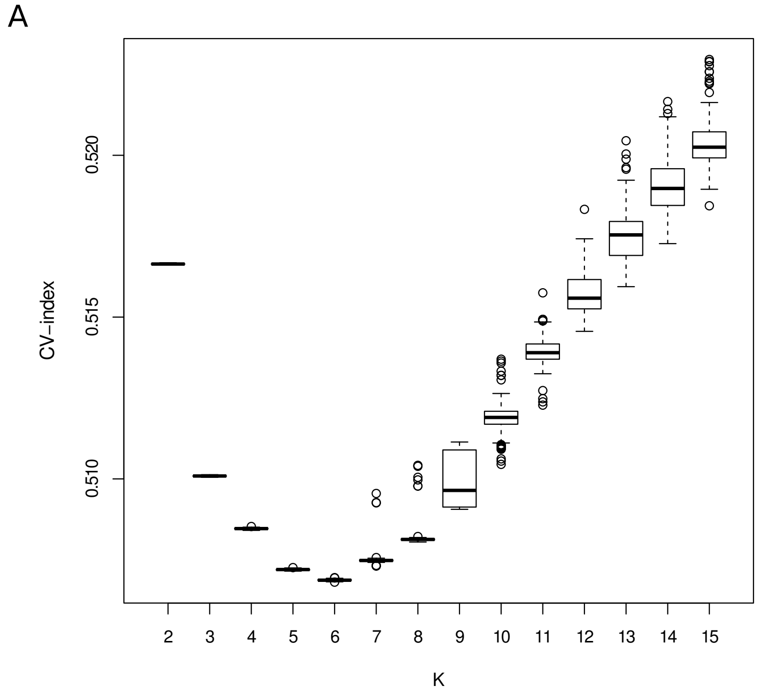


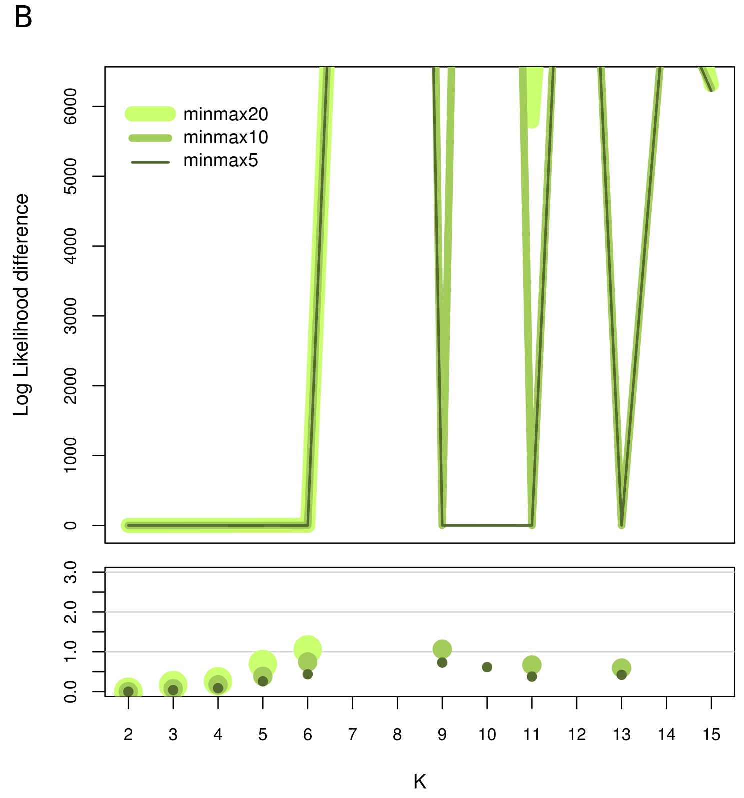


**Supplementary Figure S3.** Selection criteria for optimum level of K.
A) Box and whiskers plot of the cross validation (CV) indexes of all runs of the ADMIXTURE analysis. B) Variation in log-likelihood (LL) scores in the fractions (5%, 10% and 20%) of runs that reached the highest LL values. We assume that at a given K a global LL maximum was reached if 10% of the runs with the highest LL score showed small variation in LL scores.

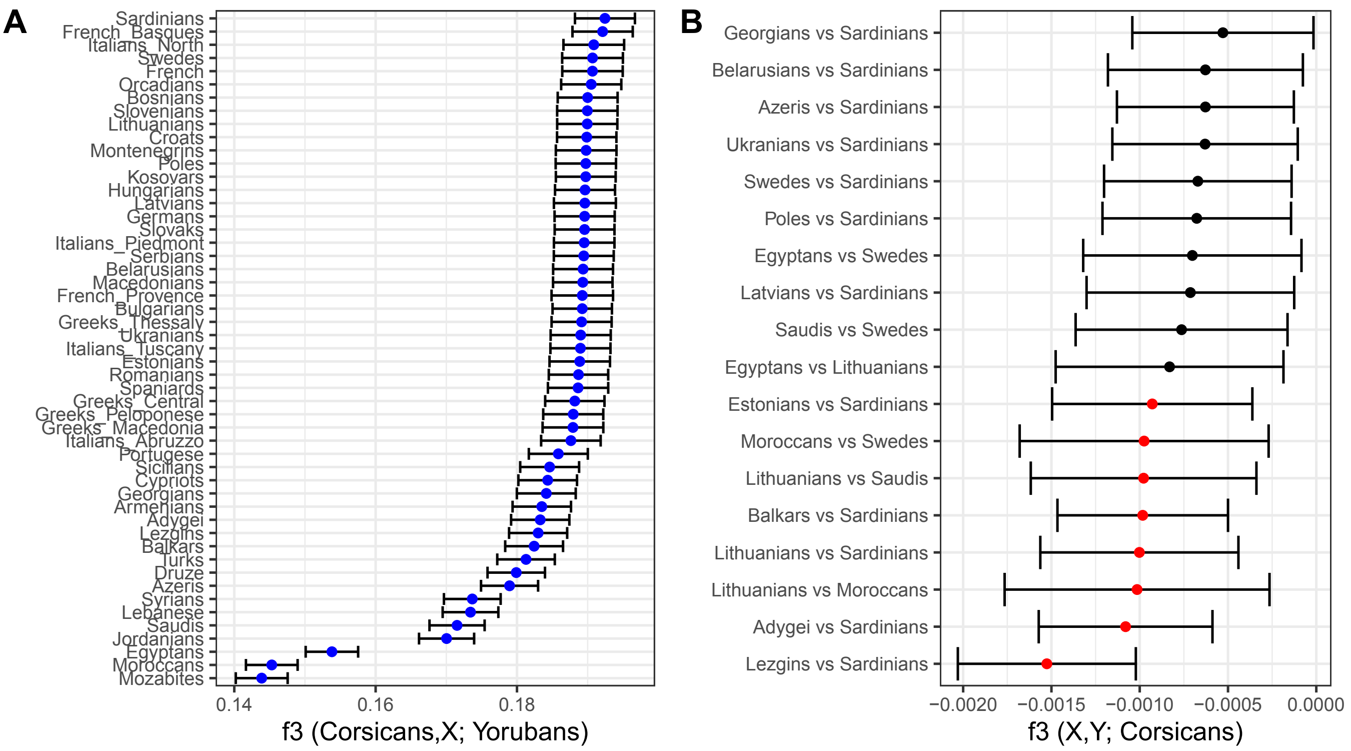


**Supplementary Figure S4.** Results of outgroup f3 and standard f3 tests.
A) Outgroup f3 results of the form f3(Corsicans, X; Yorubans) showing shared drift of Corsicans and reference populations (Supplementary Table S1) from Yoruba populations as outgroup.B) Admixture f3-statistics of the form f3(X ,Y; Corsicans), where X and Y represent all possible pairs of combinations of source populations specified in Supplementary Table S1. Black and red dots refer to |Z| higher than 3 and 4, respectively.

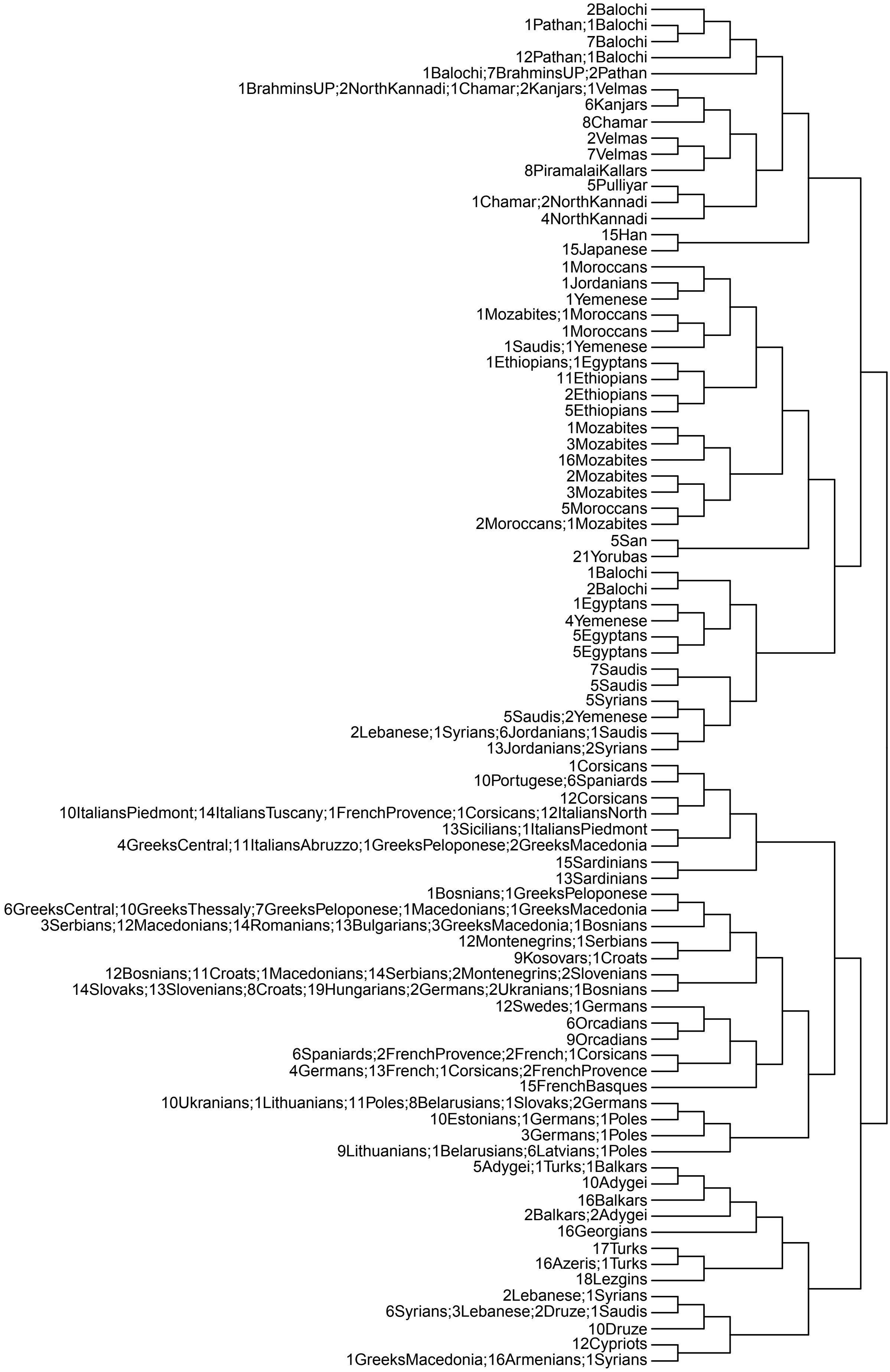


**Supplementary Figure S5.** fineSTRUCTURE dendrogram of all samples. Dendrogram clusters individuals based on similarity of copying vectors. Cluster labels refer to population name and number of individuals from this population. Correspondence with a given cluster name and a macroregional grouping is reported in Supplementary Table S3.

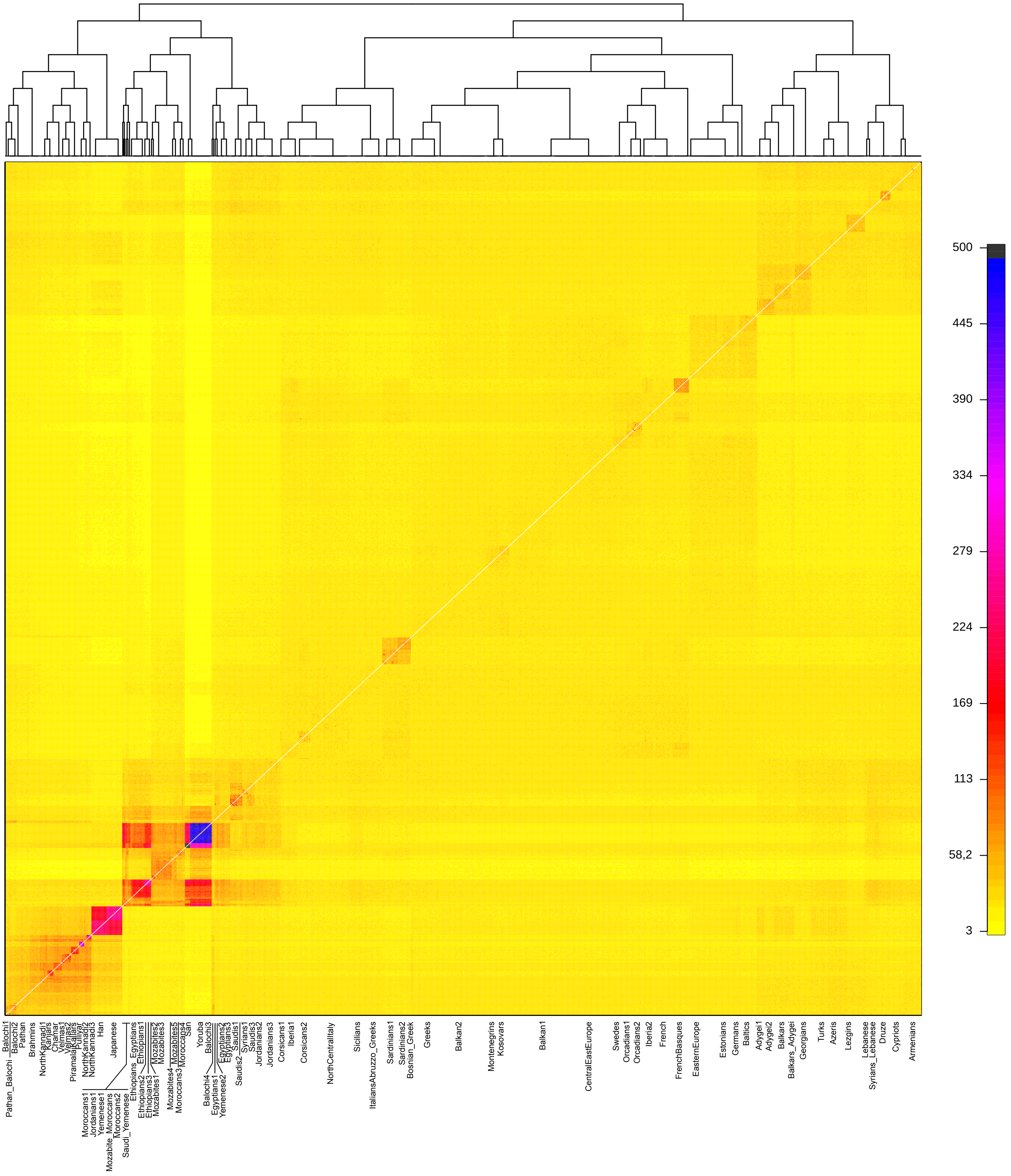


**Supplementary Figure S6.** Coancestry matrix as inferred by ChromoPainter/fineSTRUCTURE.

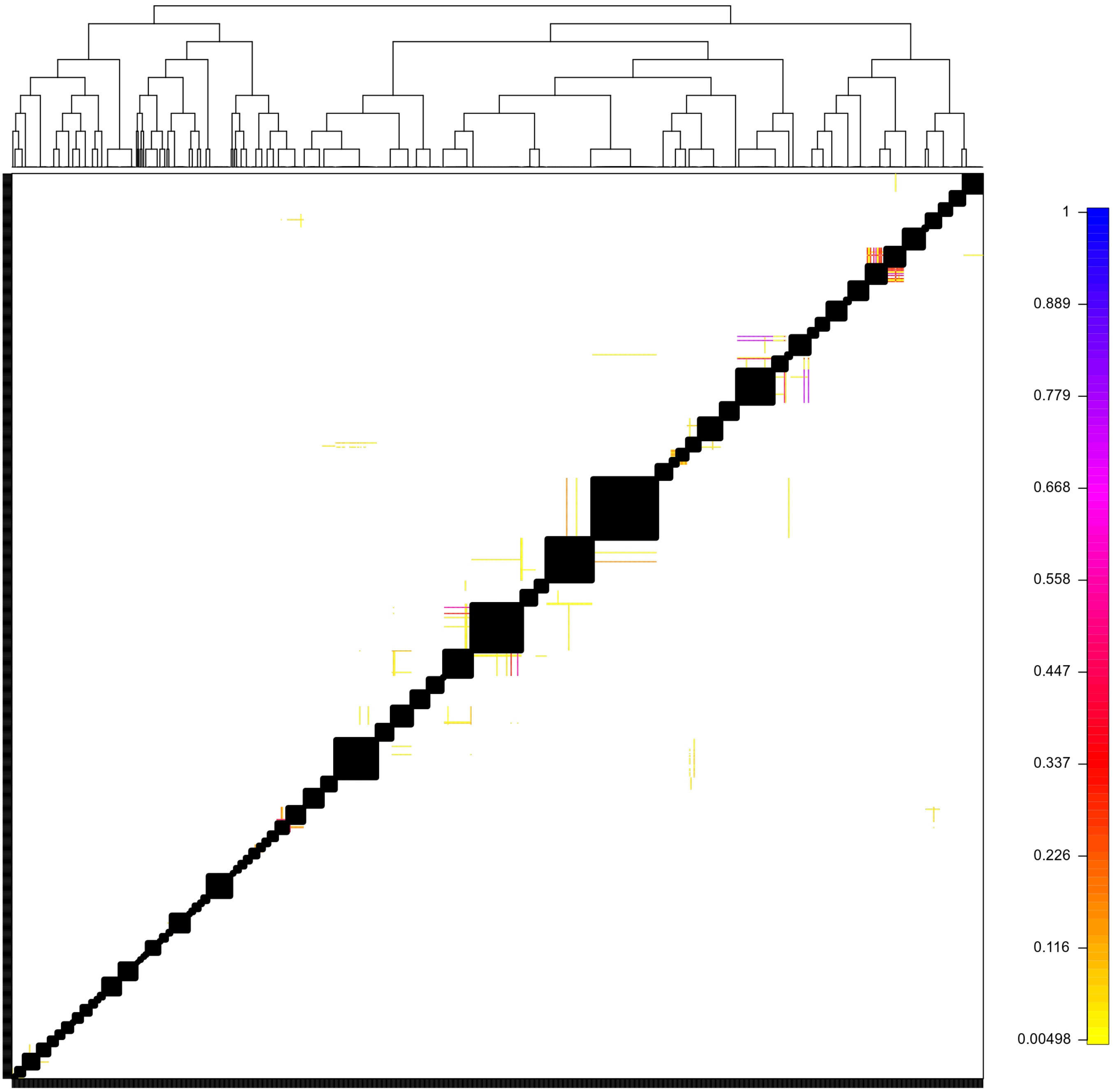


**Supplementary Figure S7.** Pairwise coincidence matrix for the ChromoPainter clustering iterations. The heatmap shows the proportion of ChromoPainter of MCMC iterations for which a pair of individuals fall into the same cluster. As expected, very high values are found at the diagonal, confirming the overall robustness of the clustering approach.

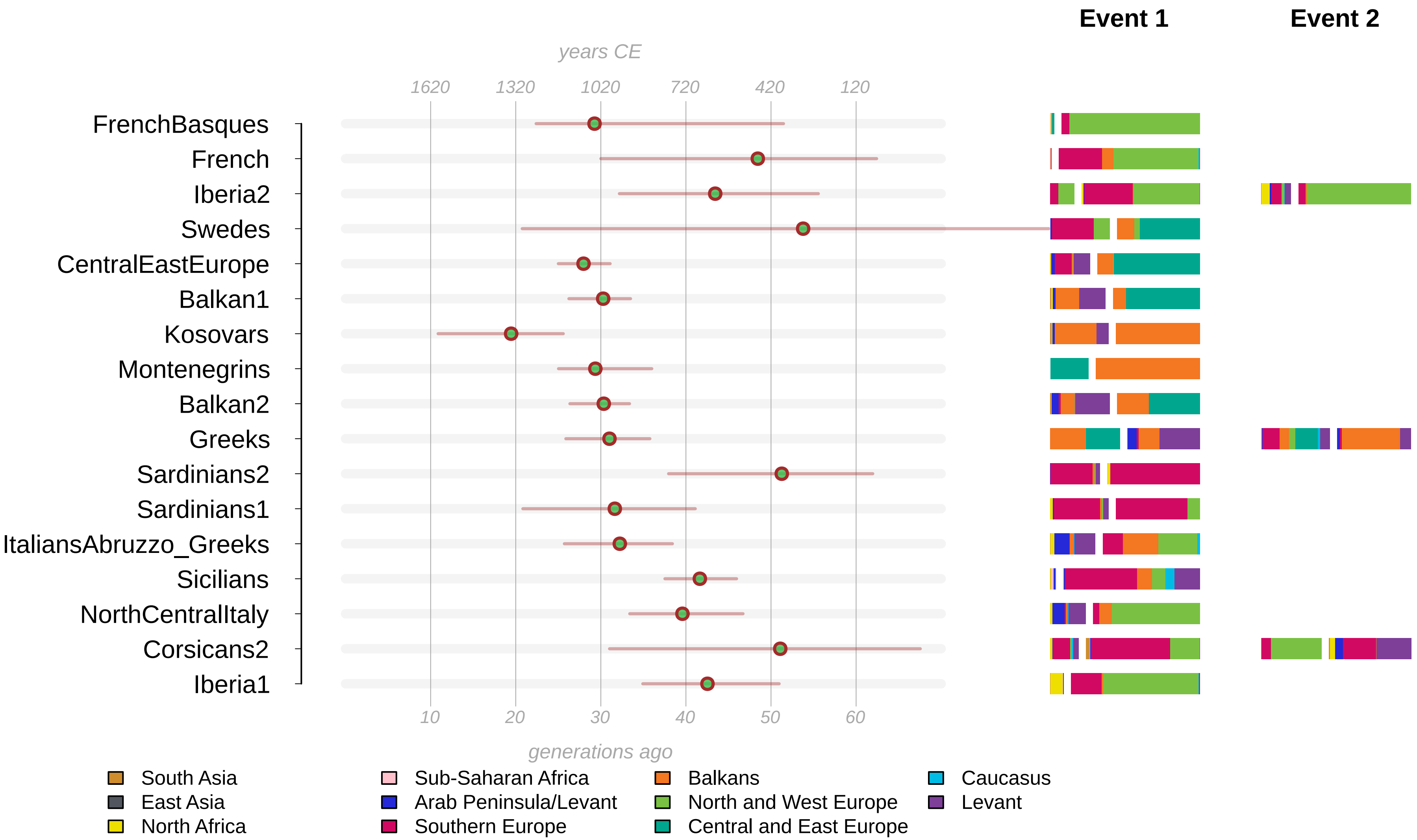


**Supplementary Figure S8.** Admixture dates as inferred by GLOBETROTTER in the "full" analysis. We fit the painting profile of Western Eurasian populations into expected curves for different admixture models, as implemented in GLOBETROTTER. The estimated dates and sources composition are shown.

**Supplementary Tables**

**Table S1. Overview of dataset composition for different performed analysis. A) Modern populations. B) aDNA samples.**

**Table S2. Pairwise $F_{ST}$ distance among analysed populations. A) FST matrix. B) Standard deviation estimates for FST.**

**Table S3. Inferred clusters by the ChromoPainter/fineSTRUCTURE analysis.** Cluster composition column indicates population name preceded by the numerosity of that population in the cluster.

**Table S4. Summary of the GLOBETROTTER results for "full" (light blue) and "non-local" (light green) analysis.**

**Table S5. qpAdm results.** List of supported four-population scenario for all the analysed populations.
